# Concomitant use of tamoxifen and endoxifen in postmenopausal early breast cancer: prediction of plasma levels by physiologically-based pharmacokinetic modeling

**DOI:** 10.1186/2193-1801-3-285

**Published:** 2014-06-05

**Authors:** Kristin Dickschen, Thomas Eissing, Thomas Mürdter, Matthias Schwab, Stefan Willmann, Georg Hempel

**Affiliations:** Institut für Pharmazeutische und Medizinische Chemie, Klinische Pharmazie, Westfälische Wilhelms-Universität Münster, Corrensstrasse 48, Münster, 48149 Germany; Computational Systems Biology, Bayer Technology Services GmbH, Building 9115, Leverkusen, 51368 Germany; Dr. Margarete Fischer-Bosch-Institute of Clinical Pharmacology and University Tübingen, Auerbachstrasse 112, Stuttgart, 70376 Germany; Department of Clinical Pharmacology, University Hospital Tübingen, Auf der Morgenstelle 8, Tübingen, 72076 Germany; Clinical Pharmacometrics, Bayer Pharma AG, Aprather Weg 18a, Wuppertal, 42113 Germany

**Keywords:** Postmenopausal breast cancer, Tamoxifen-endoxifen-combination, CYP2D6 genotype, PBPK modeling

## Abstract

**Purpose:**

To overcome cytochrome P450 2D6 (CYP2D6) mediated tamoxifen resistance in postmenopausal early breast cancer, CYP2D6 phenotype-adjusted tamoxifen dosing in patients with impaired CYP2D6 metabolism and/or the application of endoxifen, the most potent tamoxifen metabolite, are alternative treatment options. To elucidate both strategies comprehensively we used a physiologically-based pharmacokinetic (PBPK) modeling approach.

**Methods:**

Firstly simulation of increasing tamoxifen dosages was performed by a virtual clinical trial including populations of CYP2D6 poor (PM), intermediate (IM) and extensive metabolizers (EM) (N = 8,000). Secondly we performed PBPK-simulations under consideration of tamoxifen use plus concomitant increasing dosages of endoxifen (N = 7,000).

**Results:**

Our virtual study demonstrates that dose escalation of tamoxifen in IMs resulted in endoxifen steady-state plasma concentrations similar to CYP2D6 EMs whereas PMs did not reach EM endoxifen levels. Steady-state plasma concentrations of tamoxifen, N-desmethyl-tamoxifen, 4-hydroxy-tamoxifen and endoxifen were similar in CYP2D6 IMs and PMs *versus* EMs using once daily dosing of 20 mg tamoxifen and concomitant CYP2D6 phenotype-adjusted endoxifen dosing in IMs and PMs (1 mg/d and 3 mg/d, respectively).

**Conclusion:**

In conclusion, we suggest that co-administration of endoxifen in tamoxifen treated early breast cancer women with impaired CYP2D6 metabolism is a promising alternative to reach plasma concentrations comparable to CYP2D6 EM patients.

## Introduction

The efficacy of the selective estrogen receptor modulator tamoxifen, the backbone of the treatment of estrogen receptor positive early breast cancer in postmenopausal women, is based on its bio-activation to 4-hydroxylated metabolites by hepatic cytochrome P450 (CYP) enzymes (Brauch et al. 2009). Its anti-cancer activity is mainly attributed to the (Z)-endoxifen isomer with an almost identical anti-estrogenic activity compared to (Z)-4-hydroxytamoxifen (4OH-TAM) but 5- to 10-fold higher steady state plasma concentrations (C_ss_) in patients (Lim et al. 2005; Wu et al. 2009).

**Figure 1:**
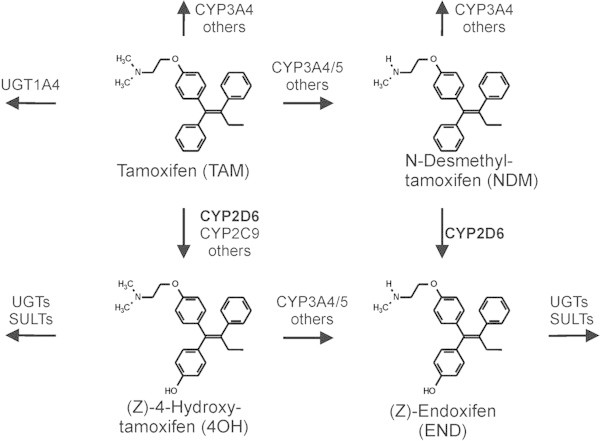
**Simplified biotransformation scheme of tamoxifen in man.** Tamoxifen is mainly N-demethylated to NDM-TAM and subsequently 4-hydroxylated to endoxifen. A minor pathway proceeds via 4-hydroxylation to 4OH-TAM followed by N-demethylation to endoxifen. The polymorphic CYP2D6 is involved in crucial steps of the endoxifen formation. *Abbreviations used in diagram: cytochrome P450 (CYP), sulfonyltransferase (SULT), uridine-5′-diphosphoglucuronyltransferase (UGT), tamoxifen (TAM), N-desmethyltamoxifen (NDM), 4-hydroxytamoxifen (4OH), endoxifen (END).*

Cytochrome P450 2D6 (CYP2D6) is highly polymorphically expressed in humans and involved in crucial steps of the formation of endoxifen [Figure 1] (Brauch et al. 2009). The majority of endoxifen arises from the primary tamoxifen metabolite N-desmethyltamoxifen (NDM-TAM) via 4-hydroxylation exclusively mediated by CYP2D6 (Desta et al. 2004). To date, more than one hundred *CYP2D6* alleles have been described (http://www.cypalleles.ki.se/cyp2d6.htm) resulting into four different phenotypes: ultra-rapid metabolizers (UM; increased enzyme activity), extensive metabolizers (EM; normal enzyme activity), intermediate metabolizers (IM; decreased enzyme activity) and poor metabolizers (PM; abolished enzyme activity). The CYP2D6 genotype-phenotype concordance rate is excellent which has been extensively studied (for review see (Zanger and Schwab 2013)). Based on clinical studies there is an increasing body of evidence that the *in vivo* metabolism of tamoxifen in postmenopausal early breast cancer depends on CYP2D6, thereby altering tamoxifen response (Brauch et al. 2013a, 2013b; Brauch and Schwab 2013). Patients stratified genetically into CYP2D6 IMs or PMs using the patient’s germline DNA showed a significant gene-dose-dependent decrease in the formation of endoxifen plasma concentrations compared with EM patients (Borges et al. 2006; Kiyotani et al. 2010; Mürdter et al. 2011; Lim et al. 2011). For instance, higher endoxifen levels correlated with a significant reduction of breast cancer recurrence rate (26%) in the Women’s Healthy Eating and Living (WHEL) trial (Madlensky et al. 2011), and IM and PM women were more likely to be in the low endoxifen bottom quintile group with an increased risk for recurrence. Moreover, data from a clinical multicenter trial support the notion that a *CYP2D6* genotype-guided tamoxifen dosing approach significantly rises endoxifen levels in IM and PM patients by doubling the daily dose from 20 mg to 40 mg. Of note, only IMs reach endoxifen levels comparable to those of EM patients receiving the standard dose (Irvin et al. 2011). Finally, direct administration of endoxifen to bypass CYP2D6-dependent bio-activation and to reduce inter-individual variability of endoxifen C_ss_ levels (Ahmad et al. 2010) may be an attractive alternative in treatment of postmenopausal breast cancer.

Physiologically-based pharmacokinetic (PBPK) modeling provides a knowledge-based approach to mechanistically describe the pharmacokinetics (PK) as well as the pharmacodynamics (PD) of drugs (Zhao et al. 2012; Eissing et al. 2011). Implementing genotype-specific enzyme activities (e.g. CYP2D6), PBPK models can be parameterized to predict the impact of different enzyme phenotypes on PK as well as PD data (Dickschen et al. 2012). Here, we present a PBPK model-based virtual clinical trial for CYP2D6 phenotype-dependent dosing of tamoxifen plus the concomitant use of endoxifen in early postmenopausal breast cancer. The virtual study aims to establish a dose algorithm providing similar tamoxifen metabolic patterns in CYP2D6 IM or PM *versus* EM patients using different dosing strategies. Ultimately, the PBPK modeling approach offers a new dimension in providing personalized treatment strategies for tamoxifen in early breast cancer with implications for future clinical trials.

## Materials and methods

### Software

The PBPK-models were constructed and coupled by means of the computational systems biology software platform including PK-Sim^®^ 4.2.4 and MoBi^®^ 2.3.5 (Bayer Technology Services GmbH, Leverkusen, Germany; http://www.systems-biology.com/products).

Population simulations were conducted using the MoBi^®^ Toolbox for MATLAB^®^ 2.2 (Bayer Technology Services GmbH, Leverkusen, Germany; http://www.systems-biology.com/products with MATLAB^®^ from The MathWorks, Inc., Natick, USA; http://www.mathworks.com/products/matlab).

### Virtual clinical trial design

The recently published and validated CYP2D6 phenotype specific PBPK model describing the formation of endoxifen via tamoxifen in European breast cancer patients was applied in a virtual clinical trial to establish model based dose algorithms for CYP2D6 IMs and PMs to achieve tamoxifen metabolic pattern comparable to CYP2D6 EM patients (Dickschen et al. 2012). Three different dosing regimens (see below) were evaluated in the virtual populations of CYP2D6 EMs, IMs and PMs (N = 1,000, each), representing the major CYP2D6 phenotypes. The results of the simulated administration protocols were compared to corresponding clinical median trough C_ss_ level taken from the literature (Mürdter et al. 2011; Madlensky et al. 2011; Irvin et al. 2011; Gjerde et al. 2010, 2008).

#### Study group A: tamoxifen standard dose regimen

The standard dose regimen of 20 mg tamoxifen once daily was simulated for a period of twelve months in all three CYP2D6 phenotype populations (N = 1,000, each).

#### Study group B: tamoxifen dose escalation

The recently published genotype-guided tamoxifen dose escalation regimen for CYP2D6 IMs and PMs (Irvin et al. 2011) was simulated in 1,000 cases, respectively. Hence, firstly we simulated four months once daily dosing of 20 mg tamoxifen followed by four months dosing using 20 mg tamoxifen applied twice daily. Secondly, four months once daily dosing using 20 mg tamoxifen followed by four months dosing of 40 mg tamoxifen once daily was assessed again in IMs and PMs. Finally, four months once daily dosing of 20 mg tamoxifen followed by four months once daily dosing using 60 mg tamoxifen in CYP2D6 PM was investigated by using the PBPK-model.

#### Study group C: tamoxifen-endoxifen fixed-dose combination

A novel treatment strategy was used combining a fixed-dose of the parent drug tamoxifen together with the different dosages of the active metabolite endoxifen in CYP2D6 IMs and PMs for a period of twelve months. The rationale behind this strategy of concomitant use of tamoxifen plus endoxifen was to achieve not only similar plasma levels of endoxifen but also of tamoxifen, NDM-TAM and 4OH-TAM in IMs and PMs comparable to CYP2D6 EM patients. In all exploratory simulations, the standard dose of 20 mg tamoxifen was kept constant and three concomitant endoxifen doses ranging from 0.5 mg to 1.5 mg once daily were investigated in CYP2D6 IMs (N = 1,000, each). In CYP2D6 PMs, four endoxifen doses ranging from 1.0 mg to 4.0 mg once daily were simulated (N = 1,000, each).

**Figure 2:**
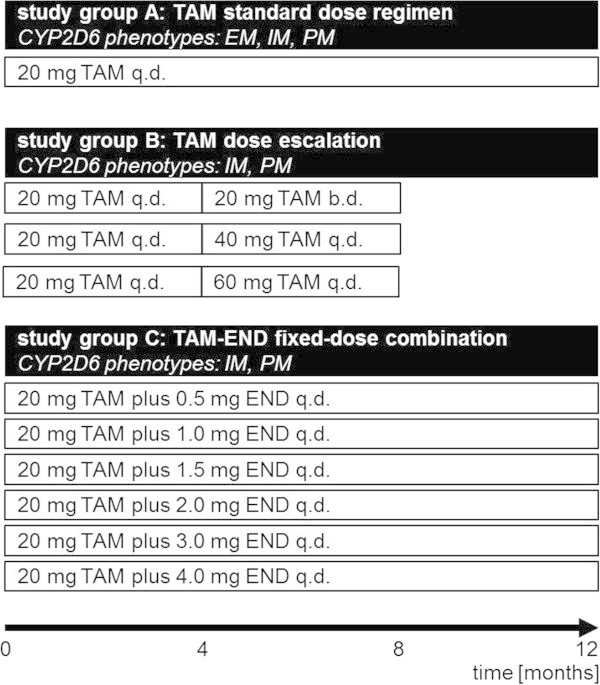
**Virtual clinical trial design.** Study group **A**: 20 mg/day tamoxifen was simulated over a period of twelve months in CYP2D6 EMs, IMs, and PMs (N = 1,000 for each phenotype group). Study group **B**: tamoxifen dose escalation to 20 mg twice daily was simulated for four months followed by four months dosing of 20 mg/day tamoxifen in CYP2D6 IMs and PMs (N = 1,000, each). Tamoxifen dose escalation to 40 mg/day was simulated for four months followed by four months dosing of 20 mg/day tamoxifen in CYP2D6 IMs and PMs (N = 1,000, each). Only in CYP2D6 PMs, tamoxifen dose escalation of 60 mg/d was simulated for four months followed by four months dosing of 20 mg/day tamoxifen (N = 1,000). Study group **C**: 20 mg/d tamoxifen plus increasing endoxifen dosages were simulated in CYP2D6 IMs and PMs for a period of twelve months (N = 1,000, each). *Abbreviations used in diagram: tamoxifen (TAM), cytochrome P450 2D6 (CYP2D6), extensive metabolizer (EM), intermediate metabolizer (IM), poor metabolizer (PM), once daily (q.d.), twice daily (b.d.), endoxifen (END).*

Thus, a total of 15 different simulation protocols for the three CYP2D6 phenotypic groups were investigated resulting in simulation data of 15,000 virtual European female early breast cancer patients. The virtual trial designs investigated are illustrated in Figure 2. From these simulations median trough C_ss_ and percentiles were calculated assuming that data are not normally distributed.

## Results

### Study group A: tamoxifen standard dose regimen

**Figure 3:**
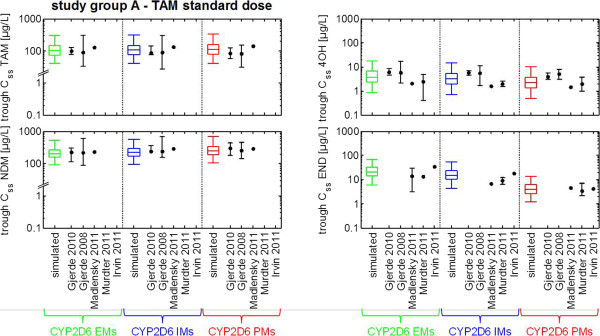
**Results of a virtual clinical trial elucidating tamoxifen standard dose regimen.** Box-whisker-plots indicate the percentiles 5, 25, 50, 75, and 95 of the population simulation results. Symbols represent CYP2D6 phenotype-specific plasma concentrations extracted from the literature of clinical trials performed in postmenopausal early breast cancer women and tamoxifen mono-therapy. *Abbreviations used in diagram: tamoxifen (TAM), N-desmethyltamoxifen (NDM), 4-hydroxytamoxifen (4OH), endoxifen (END), cytochrome P450 2D6 (CYP2D6), extensive metabolizer (EM), intermediate metabolizer (IM), poor metabolizer (PM).*

The previously developed and validated PBPK model describing the CYP2D6-dependent formation of endoxifen via tamoxifen (Dickschen et al. 2012) nicely reflects the phenotype-specific median trough C_ss_ of tamoxifen, NDM-TAM, 4OH-TAM and endoxifen in CYP2D6 EMs, IMs, and PMs [Figure 3] observed in several clinical trials (Mürdter et al. 2011; Madlensky et al. 2011; Irvin et al. 2011; Gjerde et al. 2010, 2008). In case of the CYP2D6 IMs experimental data for 4OH-TAM and endoxifen trough C_ss_ show high variability which is mirrored by our model as well. The PBPK model parameterization of the CYP2D6 IM phenotype was based on one functional *CYP2D6* allele (acc. to (Mürdter et al. 2011; Coller et al. 2002)).

Since the PBPK model adequacy is sufficiently assessed, simulated median trough C_ss_ levels of tamoxifen, NDM-TAM, 4OH-TAM and endoxifen as well as the percentiles 5 and 95 for the CYP2D6 EM phenotype were used as reference ranges to elucidate alternative dosing regimens of tamoxifen together with endoxifen in CYP2D6 IMs and PMs by PBPK simulation.

### Study group B: tamoxifen dose escalation

**Figure 4:**
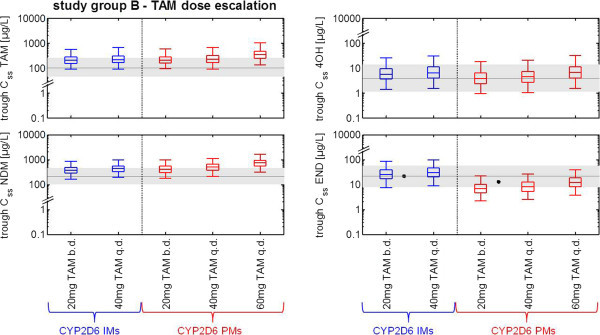
**Results of a virtual clinical trial elucidating tamoxifen dose escalation.** Box-whisker-plots indicate the percentiles 5, 25, 50, 75, and 95 of the population simulation results. Shaded grey areas represent median and the percentiles 5 and 95 of simulated reference steady-state CYP2D6 EM levels (acc. to Figure 3) as target concentration ranges for all dose escalation regimens. Corresponding plasma concentrations after dose escalation of tamoxifen (20 mg twice daily over a period of 4 months) in breast cancer patients extracted by Irvin WJ, Jr. et al. (Irvin et al. 2011) are given as symbols. *Abbreviations used in diagram: tamoxifen (TAM), N-desmethyltamoxifen (NDM), 4-hydroxytamoxifen (4OH), endoxifen (END), cytochrome P450 2D6 (CYP2D6), intermediate metabolizer (IM), poor metabolizer (PM).*

The simulation of tamoxifen dose escalation in CYP2D6 IMs results in increasing median endoxifen trough C_ss_ that reach the dimension of simulated median endoxifen trough C_ss_ in CYP2D6 EMs. These simulation data are similar to plasma levels of endoxifen quantified in EM patients in clinical trials [Figures 3 and 4]. Of note, simulated median endoxifen trough C_ss_ following 20 mg tamoxifen applied twice daily were comparable to those observed in the multicenter trial of CYP2D6 genotype-guided tamoxifen dosing using an identical dosing regimen (Irvin et al. 2011). Likewise, simulated 4OH-TAM trough C_ss_ in CYP2D6 IMs correspond to the 4OH-TAM reference range in CYP2D6 EMs. However, simulated trough C_ss_ levels of tamoxifen and NDM-TAM significantly exceed the reference values in CYP2D6 EMs [Figure 4].

Generally, tamoxifen dose escalation in CYP2D6 PMs increases the median endoxifen trough C_ss_ in the PBPK-model in line with data from a clinical study (Irvin et al. 2011). However, using the three different dose escalation regimens all simulated median trough C_ss_ of endoxifen are constantly lower compared to the 25^th^ percentile of the simulated reference endoxifen trough C_ss_ in CYP2D6 EMs (PBPK model 25^th^ percentile: 14.08 μg/L). The dose escalation regimen of 20 mg tamoxifen twice daily and 40 mg tamoxifen once daily even results in median trough C_ss_ of endoxifen (6.8 μg/L; 8.14 μg/L) that only match the 5^th^ percentile of the simulated reference C_ss_ of endoxifen in CYP2D6 EMs (7.5 μg/L). Simulated 4OH-TAM trough C_ss_ in CYP2D6 PMs regarding to both dose escalation regimens (20 mg tamoxifen twice daily: 3.9 μg/L; 40 mg tamoxifen once daily: 4.5 μg/L) are similar to the 4OH-TAM reference range (median: 3.8 μg/L) in CYP2D6 EMs, whereas trough C_ss_ levels of tamoxifen and NDM-TAM in CYP2D6 PMs exceed CYP2D6 EM reference levels up to threefold [Figure 4].

### Study group C: tamoxifen-endoxifen fixed dose combination

A standard oral dose of once-daily 20 mg tamoxifen and concomitant increasing oral doses of endoxifen were assessed by PBPK simulation. For both phenotypes, CYP2D6 IM and PM, the simulation data indicate that concomitant use of tamoxifen and endoxifen results in systemic exposure levels of tamoxifen, NDM-TAM, 4OH-TAM and endoxifen comparable to those in CYP2D6 EMs.

**Figure 5:**
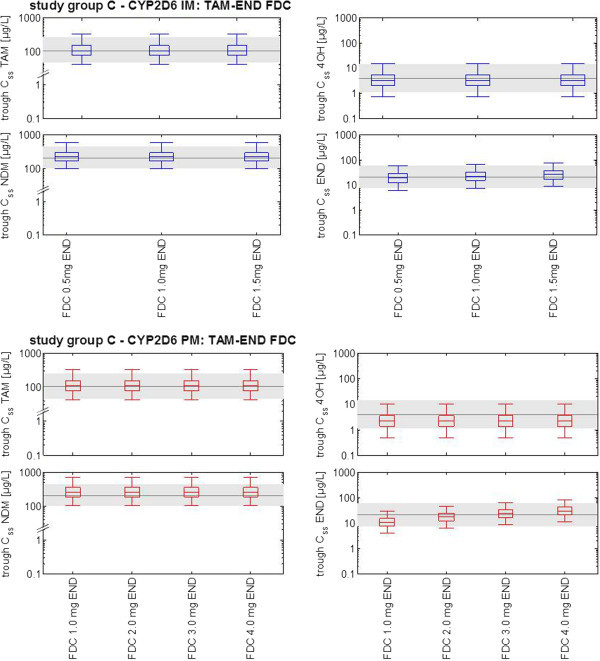
**Results of a virtual clinical trial for tamoxifen-endoxifen fixed dose combination.** Box-whisker-plots indicate the percentiles 5, 25, 50, 75, and 95 of the population simulation results. Shaded grey areas represent median and the percentiles 5 and 95 of simulated reference steady-state CYP2D6 EM levels (acc. to Figure 3) as target concentration ranges for all fixed-dose combinations. A standard dosage of 20 mg tamoxifen was used for all simulations. *Abbreviations used in diagram: tamoxifen (TAM), N-desmethyltamoxifen (NDM), 4-hydroxytamoxifen (4OH), endoxifen (END), cytochrome P450 2D6 (CYP2D6), intermediate metabolizer (IM), poor metabolizer (PM), fixed-dose combination (FDC).*

The fixed-dose combination of 20 mg tamoxifen and 1 mg endoxifen applied once daily results in almost identical median trough C_ss_ of tamoxifen, NDM-TAM, 4OH-TAM, and endoxifen in CYP2D6 IMs versus CYP2D6 EMs. In case of CYP2D6 PMs, the combination of 20 mg tamoxifen and 3 mg endoxifen reaches similar median trough C_ss_ of tamoxifen, NDM-TAM, 4OH-TAM and endoxifen as compared with simulated reference steady-state EM levels [Figure 5].

## Discussion

CYP2D6 phenotype related alteration of endoxifen plasma levels in tamoxifen treated postmenopausal early breast cancer women is well established and contributes to treatment outcome (Borges et al. 2006; Kiyotani et al. 2010; Mürdter et al. 2011; Lim et al. 2011; Madlensky et al. 2011). To overcome an increased risk for breast cancer recurrence in *CYP2D6* variant patients two different approaches have been suggested (i) CYP2D6 phenotype-adjusted tamoxifen dosage in CYP2D6 IMs and PMs to achieve similar endoxifen C_ss_ compared with EMs and (ii) direct administration of the active metabolite endoxifen which ensures independence from the CYP2D6 polymorphism.

Recently the feasibility of dose adjustment in early breast cancer women was demonstrated by several studies using increasing tamoxifen daily dosages from 20 up to 40 mg in IM and PM patients (Irvin et al. 2011; Barginear et al. 2011; Kiyotani et al. 2012). Whereas in IMs the endoxifen levels reached almost similar concentrations compared to EMs, endoxifen plasma levels in PMs were still significantly lower. This indicates that at least in PMs the concept of dose escalation of tamoxifen up to 40 mg does not seem to be an appropriate alternative in clinical practice.

The sole administration of oral endoxifen instead of tamoxifen appears to be safe and was well tolerated in a first-in-man study. Endoxifen is rapidly absorbed and systemically available as shown exemplarily for the dose range of 0.5 to 4.0 mg (Ahmad et al. 2010). Currently some clinical phase 1 trials are ongoing elucidating the use of endoxifen in adults with refractory hormone receptor-positive breast cancer, desmoid tumors, gynecologic tumors, or other hormone receptor-positive solid tumors (ClinicalTrials.gov Identifier: NCT01273168; last access date Nov 18, 2013) or in patients with metastatic or locally recurrent ER-positive breast cancer (ClinicalTrials.gov Identifier: NCT01327781; last access date Nov 18, 2013). Nevertheless, such an approach neglects the fact that intermediary metabolites like NDM-TAM and 4OH-TAM or even tamoxifen may have own anti-estrogenic activity, thereby contributing to the anti-tumoral effects of tamoxifen *in vitro* and *in vivo* (Coezy et al. 1982; Maximov et al. 2013).

Therefore, we aimed to establish robust and valid dosage algorithms by a PBPK modeling approach using different virtual trial designs to provide similar tamoxifen metabolic patterns in CYP2D6 IM or PM *versus* EM patients.

The methodology of PBPK modeling and its increasingly important role in drug development has been extensively reviewed during the past few years (Huang and Rowland 2012; Leong et al. 2012). The mechanistic and knowledge-based approach of PBPK modeling enables simulations and predictions providing insight into scenarios faster, cheaper, or easier than experimentally feasible (Edginton et al. 2006; Willmann et al. 2009). The impact of PBPK modeling in clinical decision support for selected populations are extensively outlined recently (Zhao et al. 2012; Lippert et al. 2012), including PK prediction for liver cirrhotic patients and children (Edginton and Willmann 2008; Kersting et al. 2012; Willmann et al. 2013). Furthermore, the impact of CYP2D6 pharmacogenomics on PK has been studied as well for codeine, a classical CYP2D6 substrate, using a mechanistic PBPK-model (Willmann et al. 2009; Eissing et al. 2012).

Our PBPK simulation experiments of increasing tamoxifen dosages in CYP2D6 PMs and IMs demonstrated that dose escalation of tamoxifen to 20 mg twice daily is only sufficient in CYP2D6 IMs in order to attain a median endoxifen C_ss_ comparable to CYP2D6 EM levels. These simulation data are in line with experimental data from a tamoxifen dose escalation trial in early breast cancer women by Irvine et al. (Irvin et al. 2011) corroborating the validity of our PBPK modeling approach. However in CYP2D6 PMs dose escalation of tamoxifen to 20 mg twice daily is not sufficient to reach the median endoxifen C_ss_ simulated and observed in CYP2D6 EMs. Virtual dose escalation even up to 60 mg tamoxifen once daily indicates that comparable median CYP2D6 EM endoxifen C_ss_ levels will not be achieved supporting the predominant role of CYP2D6 in the formation of endoxifen.

Although tamoxifen is well tolerated it remains unclear on whether high doses of tamoxifen may result in long-term detrimental effects like an increased risk for endometrial cancer or thromboembolic event. For instance it has been reported that high dose tamoxifen in the management of various cancers is associated with severe side effects or even increased mortality (Decaudin et al. 2004; Puchner et al. 2004; Bourla et al. 2007). Of note, our simulation data of tamoxifen dose escalation in CYP2D6 IMs and PMs indicate that plasma levels of tamoxifen as well as NDM-TAM are 3-times higher compared to levels in CYP2D6 EMs using 20 mg tamoxifen standard dosage. These simulated plasma levels are in line with data reported in patients using standard tamoxifen therapy independent from ethnicity (Mürdter et al. 2011; Lim et al. 2011; Kiyotani et al. 2012). Although the evidence is lacking that long-term side effects of tamoxifen treatment are directly linked to elevated tamoxifen metabolite levels (e.g. NDM-TAM), the strategy of dose escalation of tamoxifen, preferable in CYP2D6 IMs, needs further evaluation.

Since short-cutting of tamoxifen adjuvant endocrine therapy by direct administration of endoxifen might result in missing beneficial effects of tamoxifen itself and primary metabolites (Maximov et al. 2013), we next simulated a concomitant administration of tamoxifen standard dosage (20 mg/d) plus increasing dosages of endoxifen by the PBPK model. Using fixed-dose combinations we established an optimal dose algorithm for European female CYP2D6 IMs and PMs to achieve a comparable pattern of tamoxifen metabolite levels compared to CYP2D6 EMs. Interestingly, CYP2D6 IMs displayed similar C_ss_ of tamoxifen, NDM-TAM, 4OH-TAM, and endoxifen when receiving a fixed-dose combination of 20 mg tamoxifen plus 1 mg endoxifen once daily. In contrast 20 mg tamoxifen plus 3 mg endoxifen are required in CYP2D6 PMs to achieve similar plasma levels of tamoxifen, NDM-TAM, 4OH-TAM, and endoxifen as compared with EMs.

## Conclusion

Taken together, data from our virtual clinical trial exemplarily demonstrate how PBPK modeling can be used as a valid tool to gain deeper insight into pharmacokinetic properties of tamoxifen and its metabolites in early breast cancer patients. The simulation data indicate a novel dosing strategy of concomitant use of tamoxifen standard dosage plus CYP2D6 phenotype-adjusted endoxifen dosing as a feasible approach to overcome differences in tamoxifen metabolite levels. Thus, the tamoxifen-plus-endoxifen approach versus tamoxifen or endoxifen monotherapy offers a new avenue in treatment of early breast cancer women. Future proof-of-concept clinical trials are warranted.
